# Violence against Emergency Department nurses; Can we identify the perpetrators?

**DOI:** 10.1371/journal.pone.0230793

**Published:** 2020-04-02

**Authors:** Evelien Spelten, Brodie Thomas, Peter O’Meara, Julia van Vuuren, Anthony McGillion

**Affiliations:** 1 Department of Psychology and Public Health, Rural Health School, La Trobe University, Melbourne, Australia; 2 La Trobe Rural Health School, La Trobe University, Mildura, Australia; 3 Department of Community Emergency Health and Paramedic Practice, Monash University, Melbourne, Australia; 4 School of Nursing and Midwifery, College of Science, Health and Engineering, La Trobe University, Melbourne, Australia; Universita degli Studi di Udine, ITALY

## Abstract

**Introduction:**

Violence against health care workers is a major issue in health care organisations and is estimated to affect 95% of workers, presenting an enormous risk for workers and employers. Current interventions generally aim at managing rather than preventing or minimising violent incidents. To create better-targeted interventions, it has been suggested to shift attention to the perpetrators of violence. The aim of this study was to identify and discuss the perceptions, held by Emergency Department nurses, about perpetrators of occupational violence and aggression.

**Methods:**

Two focus groups were conducted with Emergency Department nurses at a major metropolitan hospital in Australia. In the focus groups, the nurses’ perceptions about perpetrators of violence against health care workers were identified and discussed. The results were analysed using descriptive analysis.

**Results:**

This study confirmed that violence is a major issue for Emergency Department nurses and has a considerable impact on them. Participants acknowledged that violence at work had become an intrinsic part of their job and they tend to focus on coping mechanisms. The nurses identified six overlapping groups of perpetrators and described their approach to dealing with these perpetrators. The results highlighted additional factors that impact on the occurrence and management of violence, such as the presence of security, wait times, and the triage system.

**Conclusions:**

Based on the focus groups with Emergency Department nurses we conclude that violence at work is an everyday danger for Emergency Department nurses, who feel vulnerable and recognise that it is not within their power to solve this issue given the societal component. Our conclusion is that attention needs to shift from equipping workers with tools to manage violence to the perpetrator and the development of interventions to reduce violence from targeted perpetrator groups.

## Introduction

Violence against health care workers is a major issue in health care organisations and is estimated to affect 95% of workers, presenting an enormous risk for workers and employers [1]. Violence has relevance in all workforce settings and has rural, metropolitan and international angles [2].

Violence has an impact at personal, organisational, and societal levels. On a *personal* level, violence at work has a major impact on the health and well-being of the worker. Violent incidents can result in injury and death, as well as increase the risk of Post-Traumatic Stress Injury (PTSI) for workers. Violence can profoundly disrupt workers’ lives and have serious financial implications such as lost income and increased health care costs [3–5]. For *organisations*, next to concerns for staff health and wellbeing, there is a huge economic imperative as the occurrence of violence results in lost days of work, work incapacity claims, loss of expertise, and increased costs in investment to enhance safe work environments [4, 5]. On a *societal* level, violence may result in poorer clinical care and it raises questions about our societal values and norms. Although there is societal outrage at every extreme violent incident against a health care worker, there does not seem to be a corresponding reduction in violent incidents. This raises the question whether violence against workers is becoming normalised and accepted as an everyday danger for the worker? [6, 7].

Whether it is being normalised or not, the importance of the problem is acknowledged through the many interventions that have been implemented. Some interventions take a strong stance against violence and its perpetrators, such as the ‘zero-tolerance’ approach to violence. In general, the focus of many interventions appears to be on managing violent incidents, rather than preventing or minimising them, as is evident in the almost universal training of health care workers in de-escalation techniques, possibly indicating a one-size fits all approach to managing violence [8]. Yet, there is little evidence that this, or any other interventions, have a significant impact on reducing the number of violent incidents [2, 5, 8].

To design interventions that focus more on preventing and minimising violence, we need to better understand the issues associated with the phenomenon. It has been suggested that attention needs to shift to the perpetrators of violence against health care workers [9]; a better understanding of types of perpetrators may result in a more tailored approach to these perpetrators of violence.

In this study we focussed on violence against Emergency Department (ED) nurses practicing at a metropolitan hospital in Australia that has identified reducing violence as a major priority. EDs are different from standard health care settings in a number of ways, which may impact the variation in violence they experience, and the way they deal with it. EDs have a patient population that is more heterogeneous than a mental health ward or aged care facility; nursing staff are less likely to have a previous relationship with the patient, unlike a family physician or a dialysis nurse. Additionally, patients and associates present to ED with an element of already elevated stress [10].

Furthermore, in recent years, ED presentations across Australia have increased considerably [11, 12]. Patients present to ED more readily, often because they do not have access to a family physician, or because they feel they require more specialised care. The staffing and resourcing of EDs has not always been in line with the increase in presentations [13, 14].

The aim of this study was to identify and discuss the perceptions, held by ED nurses, about the perpetrators of occupational violence and aggression. Mapping the perceived characteristics of perpetrators will inform the development of tailored interventions to reduce the risk of serious harm to health care workers. Although we acknowledge that violence against health care workers is unlikely to be completely eradicated, the ultimate aim is to design and implement interventions focusing on the perpetrator that will reduce and minimise this violence and to contribute to a safer work environment.

In this study we addressed the following research questions:

1. Do ED nurses distinguish between different categories of perpetrators?
2. How do they respond to different perpetrators profiles?

## Methods

Two focus groups were held, at the hospital, with nurses from the ED, in which the nurses’ perceptions of the perpetrators of violence against health care workers were identified and discussed. The participants were asked to identify possible categories of perpetrators, without needing to be exhaustive or discuss them in a particular order. The focus groups questions are described in Table 1, for this paper we focussed on questions 3 and 4.

**Table 1:** Focus group questions.

**Focus Group Questions**
We have four questions to ask you today. We will be discussing 2 topics: reporting of violence incidents and the perpetrator. 1. Have you ever you reported a violence incident? And what were the barriers or what was the motivation when doing so? 2. How does this work with the newly implemented system? 3. How did you perceive the perpetrator? • Common perpetrators? • Do you have a different approach to different types of perpetrators? 4. Do you think it will be possible to work with perpetrators to come to a solution to reduce the violence against health care workers? • What do you think of the new approach, the letter? Will it work?

Each focus group had a maximum of 10 participants and lasted a maximum of 90 minutes. They were audio recorded for transcription and analysis. Verbal consent was obtained and recorded at the start of the focus group. Using verbal consent procedures for focus groups has become standard practice as they are an acceptable and more efficient way to obtain consent.

The focus groups were moderated by authors ES, BT, JV and AM, two females and two males, all researchers on this project. No additional persons attended the focus groups.

The ED where this study took place assesses around 240 patients per day, or 80,000 annually– 16,000 of these arrive by ambulance. It is a Level 1 tertiary referral centre, served by a helipad and a high acuity clientele–almost half of all patients require hospital admission. It is located in the Northern tip of the Melbourne CBD, providing care both to the local community and to trauma patients across Victoria, along with another health organisation.

For the reporting of our results, we used the COREQ standard [15].

Ethical approval for the collection of data from the ED nurses and the recording of verbal consent, was granted by the hospital’s Ethics Committee under number QA2018132.

### Data collection

The hospital invited their ED nurses to participate in the focus groups. They were provided with a Participant Information Statement, which explained the purpose of the study and the role of the researchers. Participants were given the opportunity to ask questions and discuss the information with others if they wished and verbal consent was obtained and recorded at the start of each focus group. The researchers sent an executive summary of results to their contact person at ED for distribution among staff.

### Analysis

The audio recordings of the focus groups were transcribed verbatim and analysed using thematic analysis [16]. Verbal consent was transcribed separately and was not included in the transcript used for analysis. We used a phenomenological approach [17] and because of the exploratory nature of the study, inductive analysis was used as it allows for categories of perpetrators to emerge from the data without the analyst searching for specific answers [18]. The data were coded by ES, BT, JV, AM using NVivo software [19].

## Results

### Participants

The focus groups comprised 18 participants; the first group had ten participants and the second eight. No participants dropped out of the focus groups. There was a gender imbalance in the group with only two male participants, which is reflective of the nursing workforce in this setting [20]. All participants worked in the ED as registered nurses. Their experienced ranged from 0–5 years to more than 15 years, with the majority having worked less than 10 years in ED (11). Most of the participants fell in the age bracket of 30–39 years (8), and the ages of the participants ranged from 18 to 59 years.

### Categories of perpetrators

With thematic analysis, six distinct but overlapping categories of perpetrators were identified which are summarised in Table 2. Within each category, several groups of perpetrators could be identified. Table 2 summarised how the nurses would approach each type of violence. Both are discussed in more detail below.

**Table 2:** Overview of six categories of perpetrators.

Category 1: Violence or aggressive behaviour that cannot be explained by an underlying health issue	
*Description /Examples*	*Approach*
This category included a mix of patients and non-patients • Family members or bystanders • Parents of children requiring care • Copycats • Patients unfamiliar with Australian health care system • Intoxicated bystanders • Young adults in general	Participants found this category of perpetrators hard to deal with for three reasons. Firstly, the Emergency Department (ED) often has no relationship with them; they may not even know their names, which makes it harder to deal with them or to report them. Secondly, participants found these perpetrators emotionally draining and they could feel intimidated. Thirdly, they detracted from the patient and the care they needed. If the nurse could not mitigate the situation or this took too much time, security would be required. On the other hand, participants felt that a simple intervention such as a brochure explaining the ‘system’ may help with people who are unfamiliar with our health care system
*“They’re the more difficult one*, *the family members*.*”* *“But you do get the frustrated family member*, *[…] They are having a stressful time and they’re concerned about their family”*	*“It’s not just the psych patients*, *[…] it’s your family members that are frustrated that their dad’s been waiting on a trolley for twenty hours*, *which you understand but can’t do anything about*.*”* *“There is a lack of education in the community about how EDs actually work*.*”*
Category 2: Violence that is related to underlying mental health issues	
*Description /Examples*	*Approach*
This category refers to patients whose violence related to underlying mental health issues. These issues could range greatly in severity and complexity	Participants understood that for patients in the second category, their mental health issues influenced these patients’ behaviour and they took that into account in their approach. However, participants did not always feel equipped to adequately deal with these patients. Some patients would have a management plan, but the ED nurses could not always access it, and sometimes the plan was considered to be unhelpful as it influenced the nurse’s perception and approach of the patient.
*“Particularly with the mental health side of ED [Emergency Department]*, *…[we’re] quite used to a level of verbal aggression and verbal violence*.*”* *“So mental health seems to be an excuse […] to not charge people who are assaulting health care professionals*. *And that’s not an excuse*. *Because they wouldn’t tolerate it so much*, *but we have to*.*”*	*“One of our big problems is the delays in EMH [Emergency Mental Health] reviews*. *Like we can talk someone down and get them to say you’re going to be seen*. *And we can calm them down and do all this diversion-tactics*, *you know*, *nicotine patches*, *diazepam*. *But if we’re waiting 6*, *7*, *8 hours for EMH*, *then they are just going to escalate”*
Category 3: Violence is related to underlying physical health issues	
*Description /Examples*	*Approach*
Examples mentioned were the patient with a delirium, sepsis, or hypoxia	The nurses felt the only way was to diagnose correctly and provide adequate treatment.
*“They are obviously some of the rarer ones*. *That acute deliria or sepsis… because whatever disease process or illness*, *that makes them really*, *really aggressive*.*”*	*“That tolerance is very different [physical health condition] from someone that*, *for example*, *comes to you with a drug induced psychosis*.*”* *“They’re medically unwell*, *and they are expected…you know*, *the delirious patients is just not aware of what they’re on about*.*”*
Category 4: Violence that is related to addiction and substance abuse	
*Description /Examples*	*Approach*
This category also included a mix of patients and non-patients • Smokers •2. Substance abusers: alcohol •3. Illicit drug users: e.g. ice, heroin	With regards to smokers, participants would try to address the situation and call security if needed. People who are intoxicated with alcohol were difficult to deal with, as de-escalation did not always work. The most frequently mentioned way to deal with aggressive ice users was the ‘sleep and sandwich’ method—give them something to eat and let them sleep it off. The nurses commented that patients were often mortified the next day when they were told about their actions and behaviour.
*“Drugs is a big one*. *So probably ice is our main issue that we have here*.*”*	*“I feel less concerned when I’m around a true mental health patient… Whereas*, *these drug-affected*, *very different approach*.*”* *“The drug and alcohol is more physical aggression sometimes*.*”* *“My official motto there is don’t poke the bear*, *just let them sleep*. *And they wake up most of the time really quite pleasant*. *They get their sandwich and leave*.*”*
Category 5: Violence that is related to a complexity of issues	
*Description /Examples*	*Approach*
These were patients whose aggressive behaviour is related to complex issues, which will often involve but is not limited to mental health issues. • Patients with more aggravated mental health disorders, e.g. a personality disorder or an ‘antisocial’ personality, or patients with a psychosis. • Aggressive patients with dementia. • Patients with an intellectual disability or acquired brain injury that impacts their behaviour. • Patients with Asperger’s or Autism • Patients with complex issues such as an aggressive patient with mental health issues, acquired brain injury, and substance abuse	The nurses acknowledged that, similar to category three, they did not always feel equipped to deal with patients whose violence was related to complex issues. A ‘sensory trolley’ had been introduced on ED which was being used extensively.
*“The main people we have issues with are your personality disorder slash anti-social personality disorders*.*”* *“The harder ones I think now can be either the ones that have acquired brain injury*, *an intellectual disability*, *combined with the mental health and*, *you know*, *some sort of drug use*. *[*.* *.* *.*] They’re…they’re really challenging*.*”*	*“Yeah I think dementia patients are a big problem for us too*. *And there is part of me that will always feel horrible about calling a code on frail 80-year-old man*. *But they…they will not hold back sometimes*. *You can’t reason with them*.*”* *“We’re not all trained in how to look after them on a daily basis*. *So when they have their carers in*, *it’s really good*. *They can say this is how to de-escalate them*, *this is how to calm them*.*”*
Category 6: Violence that is related to repeat visitors/offenders	
*Description /Examples*	*Approach*
These were patients who visit ED frequently and were known to have been aggressive or violent during previous visits	With regards to repeat presenters, it became apparent in the focus groups that the ED nurses know their patients very well, they felt the only way to deal with these patients was to ‘speak their language’. The participants recognised that repeat presenters needed to go somewhere for their health issues, emphasising the uselessness of a ban on patients. Involving security did not always help as they would be escorted out but would probably return. These patients would often have a management plan but, as earlier stated, that could be difficult to access and could work counterproductive.
*“And I think I could name 5 names of the top of my head …they’re not deterred and they thrive on conflict*, *I suppose*. *And the police know them*, *we know them*.*”* *“A recurrent presenter that is problematic […] they usually have a management plan*. *So they’ve been here a while*, *they’re quite an easy pathway to deal with*.*”*	*“When we find that they don’t actually need to be here*, *telling them to go is a big issue*, *because a lot of people have social circumstances*. *They don’t have anywhere to go*. *They don’t have food*. *You know*, *things like that*. *So they just want a roof*.*”* *“You may have report it ten times or call the police and then they come turf them*, *then they get sectioned by another set of cops and they bring them straight back*.*”*

#### Category 1: Violence that is not related to a health issue

This diverse set of perpetrators refer to people whose violence or aggressive behaviour could not be explained by an underlying health issue. They could be patients but more often were bystanders or family members who were repeatedly referred to as persistent sources of violence. The participants suggested that family members could become violent if they feel frustrated, stressful, helpless, or entitled. ‘Copycats’ were next identified in this category, these are patients or bystanders who are under the impression that other visitors to ED ‘got away’ with having an aggressive attitude and might have received preferential treatment—they then tried the same behaviour. Next were patients unfamiliar with our health care system, travellers from overseas, or patients with a different ethnic background with a lack of English language skills or understanding of the ED system. This can lead to frustration and irritation, leading to aggression and violence. Lastly, in general, young adults were identified as a separate group with young men being aggressive/threatening and young women being ‘mouthy’ or verbally aggressive.

#### Category 2: Violence related to underlying mental health issues

Patients with mental health issues were identified as an important category of perpetrators. One of the problems is the long wait time for mental health assessments, which leaves ED nurses to manage these patients. Participants mentioned that mental health issues ranged greatly in severity and complexity and that the situation becomes more complicated when mental health issues are used as an excuse for aggressive behaviour. The complex nature of mental health presentations could have an impact on the willingness of nurses to call for help or report incidents because they did not want to aggravate the patient’s situation.

#### Category 3: Violence related to underlying physical health issues

An important category of perpetrators identified were patients who became violent because of an underlying medical condition, such as delirium, sepsis or hypoxia. The participants noted that the challenge is to determine that there is a physical health issue, and not assume it is a mental health or cognitive issue, and then identify the right issue and course of action.

#### Category 4: Violence related to substance abuse and addiction

Unsurprisingly, the ED nurses identified concerned patients or non-patients, whose violent behaviour was related to substance abuse and addiction. In this category, smokers were singled out; it was noted that their behaviour (mostly smoking where it is not allowed) and attitude (not willing to stop or move) was often so aggressive in nature that the participants considered them to a separate group of perpetrators. A recent real-life example of violence related to smoking occurred in another Organisation similar to the one where this study took place with devastating consequences[21].

In this category, several groups of substance abusers were identified. Alcohol was perceived to be a bigger problem than any other form of substance abuse in relation to violent behaviour. Illegal substance users were seen as a group more prone to violent behaviour; ICE users were described as ‘hard work’—some participants commented on how relatively ‘easy’ in hindsight heroin addicts were in the past.

#### Category 5: Violence related to a complexity of issues

A large group of patients whose aggressive behaviour is related to complex issues often involving, but not limited, to mental health issues were identified. These patients, presenting with more aggravated mental health disorders, were perceived as more complex and include patients with a personality disorder, or ‘antisocial’ personality, or patients with a psychosis. Equally, aggressive patients with dementia were challenging to deal with, as they do not ‘hold back’. Another identified group were patients with an intellectual disability or acquired brain injury that have persistent behavioural challenges as a result of their condition. The participants mentioned that patients with Asperger’s or Autism were a daily occurrence in ED. Nurses felt they were not adequately trained to deal with these patients. Some perpetrators had very complex issues, such as an aggressive patient with mental health issues, acquired brain injury, and substance abuse—they were perceived as very challenging.

#### Category 6: Violence that is related to repeat visitors/offenders

Repeat presenters/perpetrators were the final category that was identified. These were patients who visit ED frequently and were known to have been aggressive or violent during previous visits. The participants often know these patients well which, in equal measures, can be a benefit and a hindrance.

### Approach to violence and aggression

The discussion extended into approaches to violence and aggression, the second research question. Different perpetrators were approached differently, varying from the gentler ‘sleep and sandwich’ to the less gentle ‘shackle and sedation’. The range of management approaches that were mentioned in relation to the different categories of perpetrators, are summarised in Table 2.

Nurses in ED were, first and foremost, guided by their duty of care when dealing with violence at work. Nurses were proud of the fact that their hospital does not ban patients–in fact, most participants felt they had a high threshold to violence and aggression due to its frequent occurrence—they had a ‘*massive tolerance’*. At the same time, they were very mindful of patient’s stresses and would often see that as an excuse or a reason for their poor behaviour.

One of the reasons why participants said it was important that they were able to identify different groups of perpetrators is that their assessment of a type of perpetrator would inform their approach to handling the situation. The intent of the perpetrator is considered important and nurses assumed that the speed and ease with which a violent situation could be controlled is very much dependant on their effective assessment of the potential source of the violence and their capability to deal with it.

### Additional issues

The following additional issues were identified in relation to violence:

- The importance of, and reliance on, security, in many different situations.
- Despite the high tolerance for violence, participants did mention feeling vulnerable. They feared retaliation and would ask security to escort them to their car or to the tram stop after work. Some participants said they would prefer to never encounter certain patients again because of the way they had behaved. Within this context, they can struggle with the unavoidability of contact with the same patient if they are a repeat presenter.
- The process of triage within the department was acknowledged to cause a bottleneck and could create a metaphorical barrier between the patient and the care provider. This could put considerable pressure on the person in this role and could add to the build-up of tension.
- The participants identified several organisational/societal factors that they thought contributed to the incidence and nature of violent incidents:
  ○ Waiting times are seen as a source of frustration and irritation.
  ○ Cultural barriers, particularly in relation to a predominantly female workforce, could lead to aggression.
  ○ The sicker the patient, the less aggression or violence you will see.
  ○ Young and less experienced staff could be, or feel, challenged more by aggressive patients.
  ○ Doctors were seen as a potential barrier to effectively deal with aggression—participants felt that doctors were less exposed to violence and could be more forgiving. This could be perceived as being unsupportive to the frontline nurse.
  ○ Social media, the instant posting and the public ‘naming and shaming’ was considered a major issue in relation to violence and made the nurses feel very vulnerable.

## Discussion

### Main findings

This study of ED nurses in a metropolitan hospital confirmed that violence is a major issue for them and it has a considerable personal impact. All participants acknowledged that violence at work had become an intrinsic part of their job and they tend to focus on coping mechanisms. The focus is more on managing and less on preventing violence. It became evident in the conversations that violence at work has a profound impact on the participants; their recall of incidents was vivid and it was clear that violent incidents had left a strong impression on them. The general sentiment was that nobody ever started a career in health care thinking this was going to be an everyday danger at their workplace. The participants acknowledged a recent culture change in their organisation and felt that the issue of aggression and violence was now addressed more seriously and to their satisfaction.

The nurses identified six overlapping groups of perpetrators and described their approach in dealing with these perpetrators. They suggested that their assessment of the type of perpetrator impacts on how they approach and deal with incidents. The results also highlighted additional factors that impact on the occurrence and management of violence, e.g. the presence of security, waiting times, and the triage system.

### Interpretation of findings

The results of this study highlighted a number of issues around violence at work that are discussed below.

#### Different perpetrators–different approaches

One of the aims of this study was to see if participants could identify different groups of perpetrators, our assumption being that there is no ‘one-size fits all’ solution to this problem. Apart from providing tools for management of violence, this approach could provide input into the much-needed prevention and minimisation of violence. Few studies have investigated nurses' subjective perceptions of workplace violence, with the majority of research in this area focussing on quantifying workplace violence [22]. Nurses differentiating between different groups of perpetrators and varying their approach to different perpetrators has not been reported on in the qualitative literature [23, 24]. We found that the participants were readily able to distinguish between different categories of perpetrators and explain how their approach to perpetrators varied.

#### Societal issue

Nurses have learnt to accept violence as an inevitable aspect of their job. They acknowledge that there are bigger societal issues involved and it is not in their power to individually solve these issues.

The phenomenon of workplace violence has a major impact on the individual victims, the organisation and society in general, whether through economic costs, a weakening of societal trust, or a normalising of unacceptable behaviour. The extension to a societal level indicates that this is a problem that is not restricted to the hospital environment, which puts constraints on what the hospital and their staff can do to prevent and reduce violence. Society as a whole needs to own the problem if effective and acceptable solutions are to be found. The recognition of workplace violence as a societal issue is not new. Almost 25 years ago, the World Health Assembly declared violence as a major and growing public health problem [25]. The focus of interventions for workplace violence in the ED is the education of staff and response to violent incidents, there is a lack of engagement from emergency departments to address workplace violence from a societal perspective [5, 8, 23].

### Strengths and limitations of the study

We see the nurses’ willingness to talk and their frankness as a significant strength of this study. Equally, the support we received from the hospital management is testament to the organisation’s commitment to a safe workplace for their workers and to finding solutions to prevent and reduce workplace violence. Another strength of this study is that it gave voice to ED nursing staff who are the first to confront the problem within the hospital setting.

A limitation is that on the data is drawn from two focus groups with 18 ED nurses from one metropolitan hospital, which reduces the generalisability of the results. Other ED staff, such as doctors and security staff, will have different perspectives that would provide valuable insights [26]. Validation of the experiences of ED nurses and other ED staff might be gained from a comparative examination of the available documentation such as incident data known as Code Grey/Black incident documentation.

## Conclusion

Based on the focus groups with ED nurses we can conclude that violence at work is an everyday danger for ED nurses, who feel vulnerable and recognise that it is not within their power to solve this issue, given the societal component. Nurses are able to identify distinct categories of perpetrators and as a result they vary their approach to violent patients or bystanders. Our conclusion is that attention needs to shift from equipping workers with tools to manage violence, to the perpetrator and the development of interventions to reduce violence from targeted perpetrator groups.

## Supporting information

S1 Checklist (PDF)
